# Are Spatial Memories for Familiar Environments Orientation Dependent?

**DOI:** 10.5334/joc.147

**Published:** 2021-01-29

**Authors:** Adamantini Hatzipanayioti, Alexia Galati, Marianna Pagkratidou, Marios N. Avraamides

**Affiliations:** 1 Center for Tactile Internet with Human-in-the-Loop (CeTI), Dresden, Germany; 2 Unit of Lifespan Developmental Neuroscience, Faculty of Psychology, Dresden, Germany; 3 Technische Universität, Dresden, Germany; 4 Department of Psychological Science, University of North Carolina at Charlotte, US; 5 Department of Psychology, University of Cyprus, CY; 6 CYENS Centre of Excellence, Nicosia, CY; 7 Department of Psychology & Center for Applied Neuroscience, University of Cyprus, CY

**Keywords:** spatial memory, perspective-taking, virtual reality, orientation, pointing

## Abstract

In one experiment we examined the organizational structure of spatial memories for familiar environments, comparing it directly with that for unfamiliar environments. Participants in the familiar condition pointed from imagined perspectives towards objects in their own rooms and their performance was compared to that of matched controls in an unfamiliar condition who carried out the same task after studying the same rooms in immersive Virtual Reality. In both conditions, participants were faster and more accurate in pointing from imagined perspectives that were aligned with the geometry of the room (vs. not aligned), suggesting the presence of orientation-dependent representations. Whereas in the unfamiliar condition pointing performance was best along a single axis, performance in the familiar condition was about equal across all 4 orientations that were aligned with the geometric structure of the room. Moreover, performance in the familiar condition was influenced by the orientation from which participants started to preview the room prior to testing; in contrast, in the unfamiliar condition performance was not influenced by the orientation from which encoding started. This finding suggests that post-encoding situational factors (e.g., the starting orientation from which an environment is previewed) can prime the accessibility of information in well-established long-term spatial memories.

During the course of our everyday lives we carry out a variety of spatial tasks within familiar environments. We find, for example, our way to work every day and can even compute alternative routes to it when necessary, as when road closures are in effect. To execute such tasks, we rely on a spatial memory we have formed about the environment from which we can retrieve and use information as the task unfolds. We use that information to monitor our movement to the goal location, to decide toward which direction to turn while navigating, to derive a shortcut, and so on. The goal of the present study is to examine the organizational structure of the spatial memories we form about familiar environments, comparing that structure to that of spatial memories about unfamiliar environments.

Although most of our spatial activity takes place within environments we know well, the majority of past research on spatial memory is about unfamiliar environments. Studies typically require participants to first study a spatial layout and then carry out a task that relies on retrieving information about the layout from memory. A popular paradigm for investigating the structure of spatial memories is the Judgement of Relative Direction (JRD) task, which involves responding to statements of the form “Imagine being at x facing y, point to z”, where x, y, and z are objects from the memorized layout. A typical finding from studies using the JRD paradigm with small-scale unfamiliar layouts is that pointing error and response time follow a sawtooth pattern. That is, performance is best for one particular orientation and the orientations perpendicular to it (the orthogonal orientations), compared to the rest (Kelly & Avraamides, 2011; Mou & McNamara, 2002; Shelton & McNamara, 2001). This finding is commonly interpreted as evidence for an orientation-dependent spatial memory that is organized around a reference orientation. This interpretation rests on the assumption that spatial information is retrieved directly from the reference direction while additional spatial computations are needed to retrieve information from other orientations. That performance is often relatively good from orientations that are orthogonal to the reference orientation was initially interpreted as evidence for representing information, at least partially, along two axes (Mou & McNamara, 2002). Recent evidence suggests that information is represented along a single reference orientation, with performance for orientations orthogonal to it also being good due to the relatively easier spatial transformations these orientations entail (Street & Wang, 2014). Importantly, past studies have shown that the reference orientation is selected based on a variety of cues, including egocentric experience (e.g., Shelton & McNamara, 1997), salient environmental cues (e.g., Shelton & McNamara, 1997; Shelton & McNamara, 2001), instructions (Greenauer & Waller, 2008; Kelly, Costabile, & Cherep, 2018), and other situational factors such as the position of and prior knowledge about a conversational partner (e.g., Galati & Avraamides, 2013).

Past research with familiar environments has also examined whether spatial memories are orientation-dependent, albeit with less consistent results. In one experiment, Sholl (1987) had college students point to familiar campus landmarks and to familiar U.S cities while facing either north or west. Results showed that pointing was equally fast between the two facing orientations for campus landmarks, but for US cities participants were faster when they faced north than west. Sholl (1987) concluded that spatial memories for familiar environments that are experienced through navigation are orientation-free whereas large-scale environments that are only learned indirectly though maps are orientation-dependent. Similarly, Evans and Pezdek (1980) demonstrated that map learning results in a representation that is aligned with the orientation of the map, whereas learning through navigation experience results in more flexible spatial memories.

In spite of these early findings about familiar environments, more recent research indicated that memories about navigable environments can also be orientation-dependent (Frankenstein, Mohler, Bülthoff, & Meilinger, 2012; Marchette, Yerramsetti, Burns, & Shelton, 2011; Yerramsetti, Marchette, & Shelton, 2013). For example, Marchette et al. (2011), had students carry out JRD towards familiar campus locations from imagined orientations at 45° intervals, and showed that pointing error followed the typical sawtooth pattern: error was the smallest when the imagined heading was aligned with north, larger for the other three cardinal orientations (i.e., south, east, and west), and the largest for the remaining diagonal orientations. Although this finding provides strong evidence for orientation-dependence, it is not clear why there was a north-up preference. As the authors discuss, this preference could have been due to the environmental structure (buildings on that particular campus were positioned along a salient north-south axis), to navigation experience (the north-south axis was the main route of travel between the residence halls and the other buildings on that campus), or to the general convention of drawing maps with a north-up orientation.

The findings of Marchette et al. (2011; see also Frankenstein et al., 2012 and Yerramsetti et al., 2013) suggest that the organizational principles that shape spatial memories for just-experienced environments still apply when these environments become more familiar. This is noteworthy because evidence from neuroscience indicates that different neural structures are recruited for the maintenance of spatial memories following extensive experience with the environment. According to the memory consolidation theory, as spatial memories strengthen over time they become independent of the hippocampus (Squire, 1992). Although this has been challenged by recent evidence showing that hippocampal activity is present for both newly-learned environments and familiar environments that are rich in details (Patai, Javadi, Ozubko, O’Callaghan, Ji, et al., 2019), other studies have shown that retrosplenial areas are active only with familiar environments, possibly reflecting the flexible switching between egocentric and allocentric representations (Bird & Burgess, 2008). Findings that spatial memories rely on different structures as they become more familiar suggest that different processes may be involved in the encoding, maintenance, and retrieval of spatial information in familiar vs. unfamiliar environments. It is therefore important to establish, through behavioral evidence as well, whether memories for familiar and unfamiliar environments are governed by the same organizing principles.

In the present study, we revisit the issue of whether spatial memories about familiar environments are orientation-dependent, by innovating over prior research in two important ways. First, we focus on small-scale environments that are unlikely to have been encoded using cardinal directions or studied through maps. This overcomes a limitation in previous studies with campus locations (e.g. Marchette et al., 2011; Sholl, 1987) and city landmarks (Frankenstein et al., 2012), where participants could have learned the spatial layouts through a combination of inputs, including navigation and map learning. Second, we compare directly spatial performance for the same environments across participants who have extensive experience with that environment vs. others who experience the environment for the first time in the laboratory. We achieve these novelties by testing students living in university dormitories about their memories for the locations of objects in their own room (familiar condition) and compare their performance to students who study the same environments in the lab via Virtual Reality (unfamiliar condition). We assume that participants living in the dorms have created spatial memories about the arrangement of objects in their room through direct experience and from all possible perspectives in that environment. This allows us to test whether indeed these participants making spatial judgments about a highly familiar environment will exhibit orientation-free performance. Using a small-scale environment also ensures that participants in the unfamiliar condition can learn locations easily and quickly. This also ensures that the spatial representation that is constructed is organized around a single reference frame as opposed to large-scale navigable environments that afford a hierarchical organization of spatial information (Hirtle & Jonides, 1985). Indeed, past research with large-scale environments indicates that people use multiple local reference frames to represent different parts of the same environment rather than creating a single integrated representation of the whole environment (e.g., Meilinger, Riecke, & Bülhoff, 2014). Here, participants were tested about their memory of a simple environment where each location was visible from any position in the room. Past research with simple (but unfamiliar) environments indicates that memory is organized around a single reference frame (e.g., Mou & McNamara, 2002). Moreover, both groups of participants were tested at a laboratory that was remote to the dormitories, making it difficult for those in the familiar condition to know, while at the lab, how they were oriented relative to their own room (see Kelly, Avraamides, & Loomis, 2007 for evidence of sensorimotor effects in immediate but not remote environments).

All participants carried out Judgments of Relative Direction (JRDs) that entailed pointing to objects from imagined perspectives. If memories for familiar environments are organized and stored from a reference orientation just like unfamiliar environments, then we should observe a sawtooth pattern of error and/or response time for both conditions, with performance being best for one particular orientation and its orthogonal headings. In contrast, if prolonged experience with the environment renders multiple perspectives equally accessible during recall, we expect no performance advantage for a single orientation in the familiar condition.

## METHOD

### PARTICIPANTS

Sixty-four students (15 male) from the University of Cyprus participated in the experiment in exchange for 10€. Half of the participants lived in a single-occupancy room at the University of Cyprus dormitories for at least one year and were recruited through personal contacts. These participants formed the *familiar* condition. The other half were selected from the student community using an online participant pool and formed the *unfamiliar* condition. Each participant recruited through the participant pool for the unfamiliar condition was matched in terms of gender and age to a participant in the familiar condition. All participants signed an informed consent form prior to the experiment and were thoroughly debriefed afterwards about the purpose of the study.

### MATERIALS

#### Images of environments

**Figure 1:**
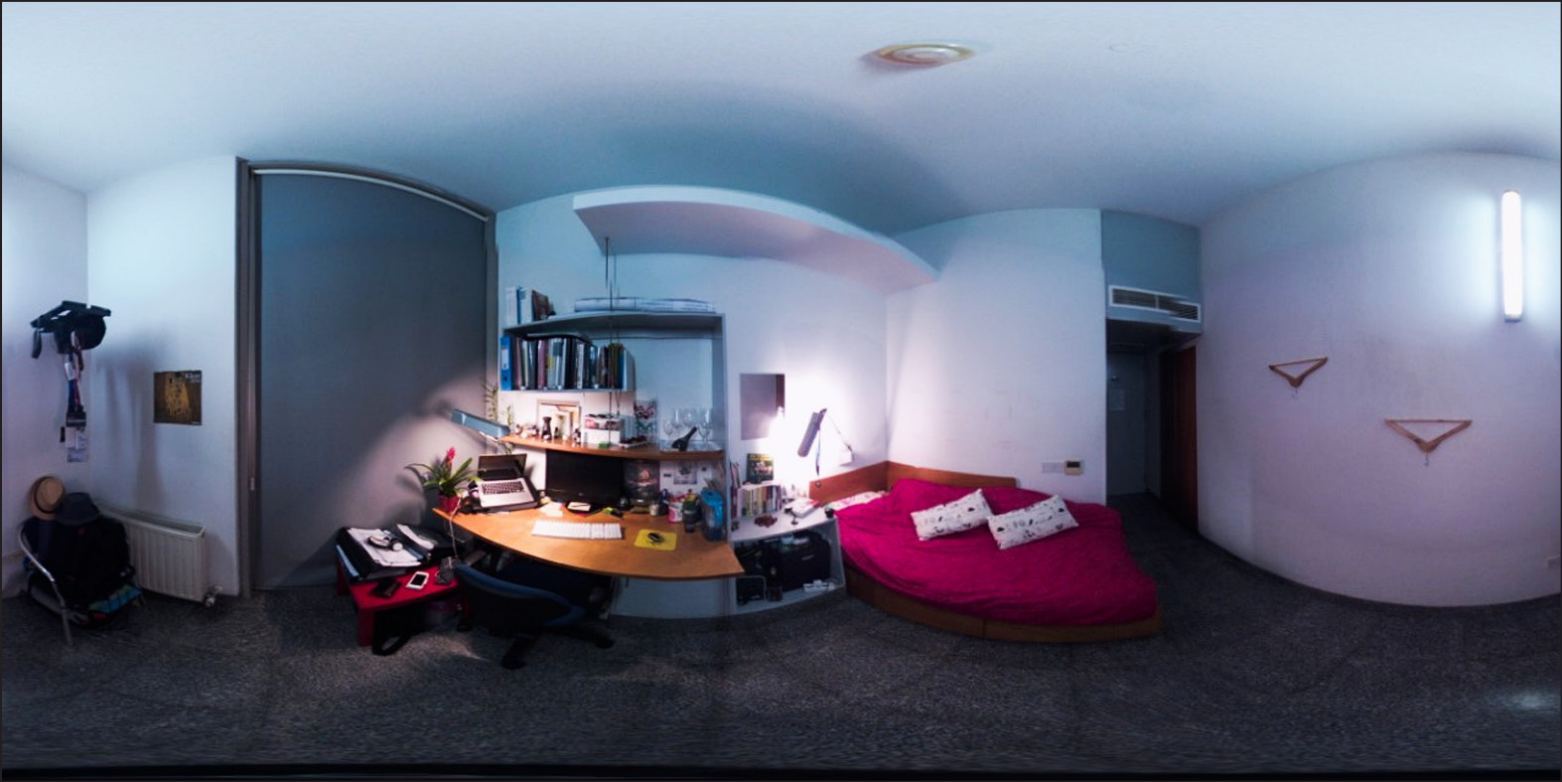
The 2D representation of a spherical image capture of a dormitory room (printed with permission from participant).

Spherical images of the rooms of students in the familiar condition were taken using a 360° spherical view camera placed in the middle of the room (see ***Figure 1***). The pictures were taken at least a week ahead of testing and at the time participants were provided with no information about how the pictures would be used in the study^1^. All rooms had the same geometric structure and were furnished. As a result, a number of objects (e.g., a wardrobe, the bed, the door etc.) were common across all rooms and at the same locations. From the pictures we were able to identify 7 to 8 objects at orientations of 45° intervals from the center of the rectangular room. Specifically, all rooms had objects located at orientations aligned with the walls of the room (i.e., 0°, 90°, 180°, 270° measured from the orientation of a person standing in the center of the room and facing a large window opposite the entrance of the room) and had at least 3 objects (and in many cases 4) at the diagonal orientations of 45°, 135°, 225°, and 315°. The spherical images that were captured could be displayed on the desktop and in a VR Head-Mounted-Display using the Kolor Eyes 360° video player.

The images of the rooms were viewed as an immersive spherical environment in an Oculus Rift Head-Mounted-Display by participants in the unfamiliar condition, and briefly on a desktop computer by participants in the familiar condition. This methodological decision served the following purposes: (1) to ensure that participants in the unfamiliar condition encoded the environment through an immersive experience, much like participants in the familiar condition previously did in their dorms, (2) to confirm, during the brief desktop-based viewing, that the object locations and object labels we used on JRD trials were consistent with the memory and object naming practices of participants in the familiar condition, (3) to ensure that participants in the familiar condition relied primarily on their memory during the JRD task, as opposed to a spatial representation created from extended exposure to their room in the laboratory.

#### Judgments of Relative Direction (JRD) task

Memory of the environments was tested with Judgments of Relative Direction (JRD) in which participants imagined adopting an orientation by facing a specified object and point with a joystick towards the direction of another object. An OpenSesame (Mathôt, Schreij, & Theeuwes, 2012) script was used to control the presentation of the stimuli and collect participants’ pointing responses and latency. The experimenter input the names of the objects determined for each participant in the script, which then produced all combinations of objects as facing orientations and as targets. Prompts had the form “Imagine facing x, point to y”, where x and y were objects from the set of identified objects for each participant.

For participants who had objects at all orientations, there were 56 unique trials (7 pairwise combinations of the 8 objects with each object serving as the facing orientation and every other object as a target). These trials were presented twice, across two blocks, but without any break or other signal to indicate a shift between blocks. For participants who had a missing object at one of the orientations, an “x” was presented in the prompt rather than an object name; participants were instructed to skip those trials. Participants used a joystick that was placed in front of them to point to each target object from the imagined heading of the instruction.

### DESIGN

The experiment followed a mixed 2 (environment: familiar vs. non-familiar) × 8 (imagined perspectives: 0°, 45°, 90°, 135°, 180°, 225°, 270° and 315°) design with imagined perspective manipulated within participants and environment between. Perspectives were labelled by starting with 0°, which was assigned to the perspective of facing the large window (i.e., the orientation one had upon entering the room) and moving clockwise. Twenty (out of 32 participants per condition) had no object on the 135° orientation. Another two participants had no object on the 225° orientation.

### PROCEDURE

Participants were tested in a laboratory located 4.2 km away from the dormitories. Those in the familiar condition were tested at least a week after the picture of their room was taken, whereas those in the unfamiliar condition were tested as they signed up, once the room picture of the participant with whom they were matched was available. Prior to the main task, all participants completed 6 practice JRD trials to familiarize themselves with the pointing task. These trials involved landmarks from the campus (e.g., “you face the campus cafeteria, point to the lab”).

Upon completing the practice trials, participants in the familiar condition were shown a spherical view of their room on the computer screen. The experimenter used a mouse to rotate the view around the room and pointed out the objects that were selected for the JRDs. The first view participants got of their room was oriented along one of the orthogonal axes of the room: 0°, 90°, 180°, or 270°. Eight participants were assigned to each *starting orientation*.

Participants in the unfamiliar condition were immersed in one of the rooms in VR with the same starting orientation as that of the participant in the familiar condition with whom they were matched. They were allowed to rotate freely to inspect the room and they were allowed unlimited time to memorize the locations of the objects. The objects were pointed out verbally by the experimenter as they came into the participant’s view. The experimenter monitored the participant’s view on a computer screen, which mirrored what was presented in VR.

Once participants indicated their readiness, they proceeded to the JRD testing that was carried out on a desktop computer. These trials involved responding to statements of the form “you are facing the door, point to the bed” for all possible combination of objects, using a joystick. Participants who did not have any missing orientations (i.e., had objects at all orientations) carried out a total of 112 trials. Those participants who were missing one orientation (due to the lack of object in the environment at that orientations) carried out a total of 84 trials (42 trials per block). Participants with missing orientations were told to ignore trials that had an “x” in the statement and to proceed to the next trial. Within a block, trials were presented in a different randomized order for each participant. Participants were instructed to respond as fast as possible but without sacrificing accuracy for speed.

Following the JRD testing, participants filled out a short questionnaire that collected demographic data such as age, gender, year of study at the university, as well as information about how long they have been living in their current room, and how much time they typically spent in it per day. This information allowed us to ensure that participants in the familiar condition lived the dormitory room for at least a year and that they spent sufficient time in it. For the unfamiliar group demographic information was also collected during recruitment and used for matching. After participants filled out the questionnaire, the experimenter asked participants to imagine standing in the middle of the room they were tested about and name the object that was in front of them. We took this spontaneously imagined perspective to indicate participants’ preferred orientation. This question allowed us to examine whether the participants’ reported *preferred orientation* was influenced by the starting orientation we had assigned them to and whether it was consistent with their testing performance.

## RESULTS

### OVERVIEW OF STATISTICAL MODELS

Data were analyzed with linear mixed-effects models, which were fitted using the lme4 package (Bates, Mächler, Bolker, & Walker, 2014) in R (R Core Team, 2020). We followed the recommendations of Barr, Levy, Scheepers, and Tily (2013) to specify maximal models with a full random effect structure, as long as the model converged.

For both models with pointing error and pointing latency as the dependent measure, we entered, as fixed effects, the environment condition (familiar vs. unfamiliar) and imagined perspective (0°, 45°, 90°, 135°/225°, 180°, 270°), along with their interaction. We combined the trials with 135° and 225° as the imagined heading into a single level because many participants were missing an object at 135° and a few at 225°, under the rationale that both perspectives deviate equally from 0°. Fixed effects were contrast coded for environment (familiar = –.5, unfamiliar =.5) and for imagined perspective (using a simple contrast with 0° as the reference category).

In follow-up analyses reported in the final two subsections of Results, we also explore models with perspective type as fixed effect, instead of the specific imagined perspective of that trial. The goal of those exploratory analyses was to assess—for familiar and unfamiliar environments, separately–the relative advantage (if any) of the participant’s self-reported preferred orientation relative to their starting orientation and all other orthogonal orientations. To do so, we defined the perspective type of each trial relative to the participant’s starting orientation and their self-reported preferred orientation. We explored separate models for the subset of data where the participant’s preferred orientation was the same as their starting orientation (i.e., involving a distinction between trials from the preferred/starting vs. other orthogonal orientation) and for the subset of data where the participants’ preferred orientation was different from their starting orientation (involving a distinction between trials from the preferred vs. starting vs. other orthogonal orientation), in familiar and unfamiliar environments respectively (i.e., a total of four models). In these models, perspective type was modelled as a fixed effect. When the participants’ reported preferred orientation was the same as the starting orientation, perspective type was contrast coded as: preferred/starting = –.5, other orthogonal =.5. When the preferred orientation was different than the starting orientation, perspective type was contrast coded across the levels of “preferred”, “starting”, “all other orthogonal” perspectives with “starting” as the reference category.

The random effect structure of the models included intercepts for participants and a random slope for perspective (imagined perspective or perspective type, depending on the analysis), to account for between-participant variation of its effect. If a model failed to converge, we simplified it by removing the random slope for heading from the random effect structure (following the recommendations of Bates, Kliegl, Vasishth, & Baayen, 2015).

The *p*-values were obtained from the lmerTest package (Kuznetsova, Brockhoff, & Christensen, 2018) using the Satterthwaite’s method. Captured variance of overall models is reported as Conditional R^2^ variance explained by fixed and random factors together, which was computed using the MuMIn R statistical package (Johnson, 2014). When visual inspection of residual plots for the models revealed deviation from normality and homoscedasticity, the dependent variable was log-transformed. That was the case for all pointing latency models.

Trials on which pointing latency was over 3 standard deviations for that participant were excluded, as well as trials with pointing error exceeding 90° since these errors could indicate object mix-up or some unsystematic disorientation. In total 398 trials were excluded, amounting to 6.8% of the data.

De-identified raw data files, along with code for preparing the data, specifying planned contrasts, and testing the statistical models, are available through our OSF repository for the project (*https://osf.io/9ags5/*).

### POINTING PERFORMANCE FOR ENVIRONMENT TYPE AND IMAGINED PERSPECTIVE

We first analyzed linear mixed effects models with environment, imagined perspective, and their interaction as fixed effects to examine (1) whether there was a difference in performance across the familiar and unfamiliar environments, (2) whether pointing error exhibited the sawtooth pattern that is typical of JRD tasks, revealing the facilitation of orthogonal perspectives, and (3) whether the predicted sawtooth pattern would be comparable in both types of environments.

**Figure 2:**
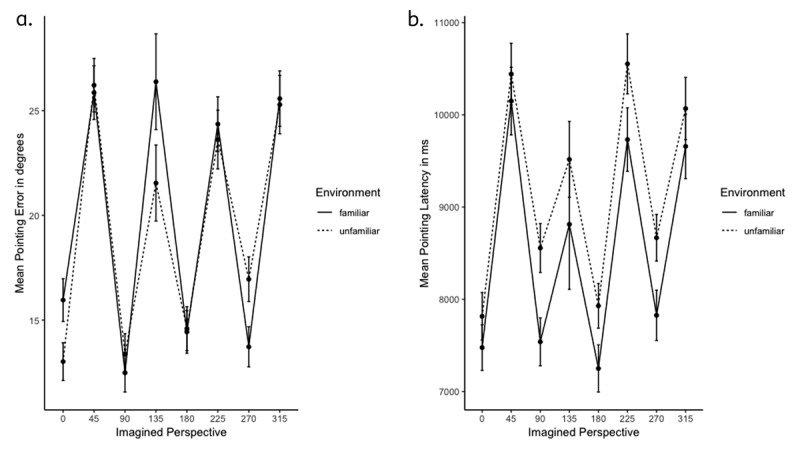
**a.** Pointing error as a function of environment and imagined perspective. Error bars represent standard errors of the mean. **b.** Pointing latency as a function of environment and imagined perspective. Error bars represent standard errors of the mean.

As shown in ***Table 1***, the environment did not significantly predict pointing error or pointing latencies. The imagined perspective that participants adopted in a given trial impacted both measures of performance: including imagined perspective in the linear mixed effects models significantly improved model fit for both pointing error (χ^2^(39) = 668.25, *p* < .001) and pointing latencies (χ^2^(11) = 61.52 *p* < .001). As seen in ***Figure 2a*** and ***2b***, pointing error and pointing latencies exhibited a sawtooth pattern, being lower for orthogonal orientations (i.e., perspectives aligned with the walls of the rooms) than those that were diagonal to them. Participants responded faster and more accurately from orthogonal than diagonal perspectives. This was corroborated by the linear mixed effects models, as performance on both measures was significantly different on trials involving the diagonal perspectives (45°, 135°/225°, 315°) compared to 0° (see ***Table 1***).

**Table 1:** Mixed-effects models for pointing error and pointing latency (log-transformed).


PREDICTOR	POINTING ERROR				POINTING LATENCY			
	
	R^2^ = .32				R^2^ = .30			
	
	B	SE	t	p	B	SE	t	p

Intercept	**19.30**	**1.30**	**14.88**	**<.001**	**8.91**	**0.03**	**64.82**	**<.001**

environment: familiar vs. unfamiliar	–0.60	2.59	–0.23	0.82	0.11	0.07	1.60	0.11

perspective: 0 vs. 45	**11.88**	**1.38**	**8.63**	**<.001**	**0.27**	**0.04**	**7.54**	**0.00**

perspective: 0 vs. 90	–1.84	1.13	–1.63	0.11	0.05	0.03	1.66	0.10

perspective: 0 vs. 135/225	**10.03**	**1.23**	**8.18**	**<.001**	**0.23**	**0.03**	**6.65**	**0.00**

perspective: 0 vs. 180	0.21	1.14	0.18	0.86	–0.02	0.03	–0.57	0.57

perspective: 0 vs. 270	0.93	1.00	0.93	0.35	**0.07**	**0.03**	**2.81**	**0.01**

perspective: 0 vs. 315	**11.27**	**1.48**	**7.59**	**<.001**	**0.23**	**0.03**	**7.68**	**<.001**

environment: familiar vs. unfamiliar * perspective: 0 vs. 45	2.33	2.75	0.85	0.40	–0.03	0.07	–0.37	0.71

environment: familiar vs. unfamiliar * perspective: 0 vs. 90	3.76	2.26	1.66	0.10	0.06	0.06	0.97	0.33

environment: familiar vs. unfamiliar * perspective: 0 vs. 135/225	0.15	2.45	0.06	0.95	0.07	0.07	1.06	0.29

environment: familiar vs. unfamiliar * perspective: 0 vs. 180	3.04	2.29	1.33	0.19	0.05	0.07	0.72	0.47

environment: familiar vs. unfamiliar * perspective: 0 vs. 270	**6.21**	**2.00**	**3.11**	**<.001**	0.06	0.05	1.26	0.21

environment: familiar vs. unfamiliar * perspective: 0 vs. 315	2.24	2.97	0.75	0.45	–0.01	0.06	–0.23	0.82


*Note*: Each dependent measure is modelled as a function of the centered and contrast coded predictors: environment (familiar = –0.5, unfamiliar = 0.5), imagined perspective (with levels 0°, 45°, 90°, 135°/225°, 180°, 270°, defined as a single contrast with 0° as a reference category) and their interaction, using the maximal random effect structure possible. For each fixed effects and interaction, we report the unstandardized coefficient and its standard error, along with the associated t-value and p-value, and the overall variance captured by the model (R^2^). Statistically significant predictors (at the *alpha* = .05 level) are in bold.

Notably, although the sawtooth pattern was present for both environment conditions, the performance decrement of diagonal perspectives depended on the environment’s familiarity, as suggested by an interaction between environment × imagined perspective. Including this interaction term in the models significantly improved model fit for pointing latencies (χ^2^(33) = 149.49 *p* < .001) and marginally so for pointing error (χ^2^(5) = 10.93, *p* = .05).

### INVESTIGATION OF THE ROLE OF PARTICIPANTS’ SELF-REPORTED PREFERRED ORIENTATION

The sawtooth pattern illustrated in ***Figure 2a*** and ***2b*** suggests an overall advantage for orthogonal orientations. The pattern of performance for the unfamiliar condition replicates what is typically reported by studies with unfamiliar environments, i.e. best performance for a single reference orientation and relatively good performance for the directly opposite orientation. The overall pattern of performance for the familiar condition–showing facilitation for all orthogonal perspectives–leaves some open questions. It could be due to participants representing the familiar environment from two reference directions or it could have arisen by different participants exhibiting best performance from a different orthogonal perspective. To investigate this possibility, we considered participants’ self-reports about the perspective they had indicated adopting when we asked them to imagine themselves back in the environment after the JRDs.

Through these follow-up analyses, we first wished to investigate whether participants’ self-reported *preferred orientation* was influenced by their *starting orientation* (i.e., the first orientation experienced upon entering the virtual environment in the unfamiliar condition and the first orientation implied by communicating the objects to be tested in the familiar condition). Therefore, in the next section, we evaluate the probability of adopting the starting orientation as the preferred orientation in the familiar and unfamiliar conditions. The prediction is that those participants reasoning about an unfamiliar environment would be more likely to adopt their starting orientation as their preferred, compared to those reasoning about a familiar environment who may have a more stable preferred perspective.

Second, we wished to establish whether the self-reported preferred orientation was indeed associated with the best performance in the pointing task. The rationale is the following: if participants in the familiar condition in fact represent the familiar environment from multiple perspectives (i.e., the comparable facilitation we observed in pointing accuracy and latency for all 4 orthogonal was not an artefact of the aggregated preferences of different individuals), then it could be that participants’ self-reported preferred orientation is not necessarily the best performing one. Toward that end, we examine whether performance in the two environment conditions was better for the preferred orientation and/or the starting orientation (in cases where the two differed) compared to other orthogonal orientations.

#### Reporting the Starting Orientation as the Preferred

Eleven out of 32 participants in the familiar condition (34%) reported the starting orientation as their preferred. This number included 7 out of the 8 participants whose starting orientation was towards the window of their room, i.e., what we had labelled as the 0° perspective. Overall, 21 out of 32 participants in the familiar condition (66%) reported a preferred orientation that was aligned with 0°, suggesting that for the majority the starting orientation did not influence their reported preferred. Instead, the perspective most participants adopted spontaneously was determined by environmental features (e.g. the large window) and/or experiential factors (e.g., the view participants had every time they entered their room). Indeed, more than half of participants (14 out of 24) who had starting orientations aligned with a perspective other than 0°, still reported 0° as their preferred.

The distribution of perspective preferences was different in the unfamiliar condition where 21 out of 32 participants (66%) reported their starting orientation as their preferred. Furthermore, only 14 out of 32 participants (44%) reported a preferred orientation along 0° and that included all 8 participants who had 0° as their starting orientation.

A chi-square test verified that compared to participants in the familiar condition, those in the unfamiliar condition were more influenced by their starting orientation in choosing what perspective to adopt when asked to imagine themselves back in the room. The distribution of preferred orientations as starting (vs. not) was significantly different across the two environments, χ^2^ (1) = 6.25, *p* = 0.012, *N* = 64.

#### Pointing Performance Based on Self-Reported Preferred Orientation

We sought to establish whether there was a performance advantage for the self-reported preferred or starting orientation in either environment condition. To do so, we compared the pointing error and the pointing latency for these orientations compared to the averaged pointing error/latency on other orthogonal orientations. Since we have already established that performance on orthogonal orientations is better than on the diagonals, we focus participants’ relative performance across these distinctions: the starting orientation (which was always an orthogonal orientation), the preferred orientation (which was an orthogonal orientation for all but one participants), and the remaining orthogonal orientations.

##### Familiar Environments

**Figure 3:**
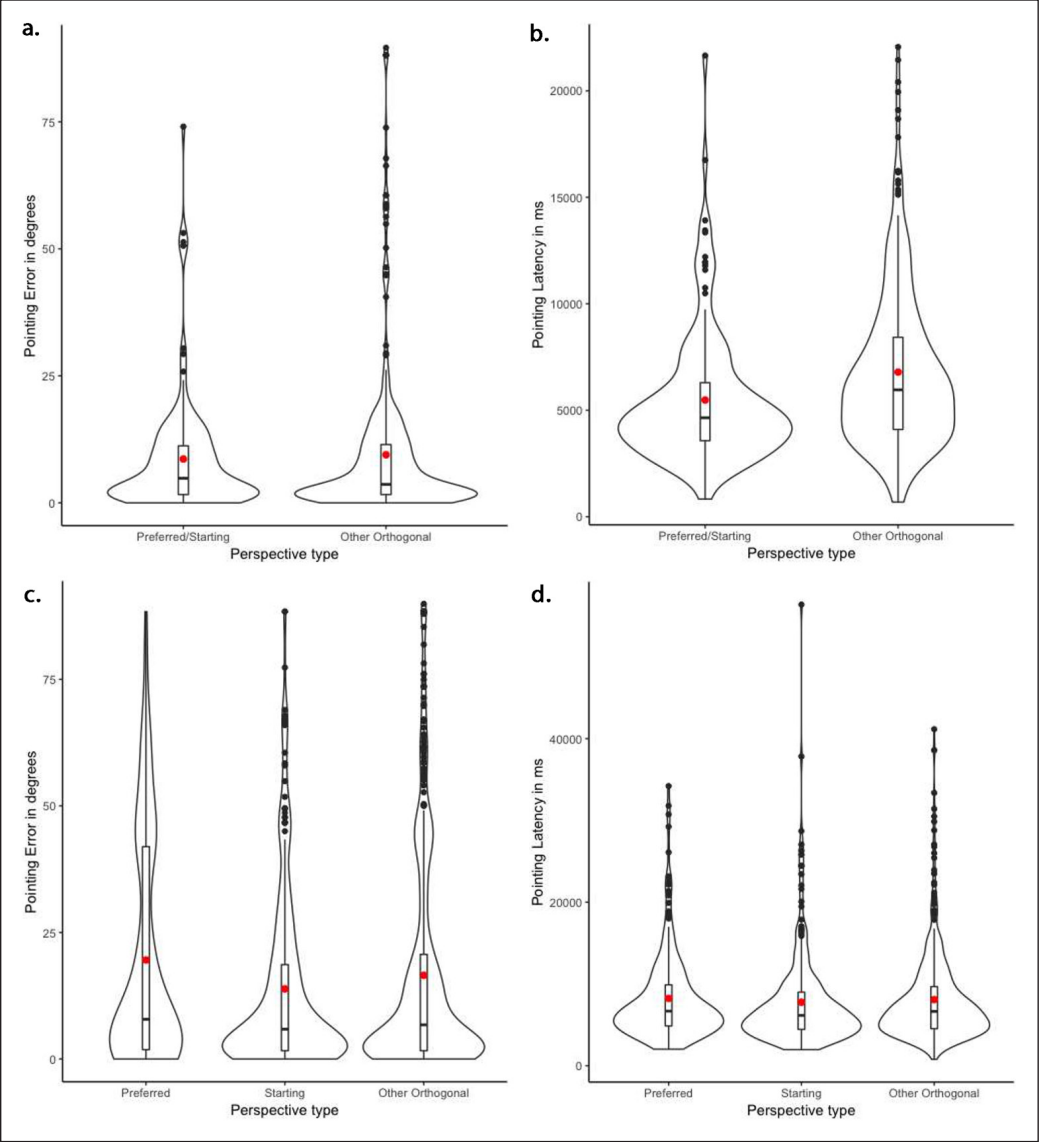
Violin plots representing the distributions of the pointing performance of participants in the Familiar environment condition. **a.** and **b.** illustrate the pointing error and latency of participants whose preferred perspective was the same as the starting perspective (*N* = 11), according to perspective type (preferred/starting vs. other orthogonal perspectives). **c.** and **d.** illustrate the pointing error and latency of participants whose preferred perspective was different from the starting perspective (*N* = 21), according to perspective type (preferred vs. starting vs. other orthogonal perspectives). Boxplots represent the median and quartiles (Q1, Q3), and the red dot represents the mean of each condition. Black dots indicate observations with values greater than Q3 plus 1.5 times the interquartile range.

First, we focused on the performance of participants in the familiar condition who reported having their starting orientation as their preferred (*N* = 11). As shown in ***Table 2***, the model for pointing error revealed that accuracy did not significantly differ from the preferred/starting orientation (*M* = 8.63°, *SD* = 11.61°) compared to other orthogonal orientations (*M* = 9.46°, *SD* = 14.72°, see ***Figure 3a***). However, the model for pointing latency revealed that the two perspectives were significantly different: participants were faster to respond from the preferred/starting orientation (*M* = 5479.50 ms, *SD* = 3191.37 ms) than the other orthogonal orientations (*M* = 6791.55 ms, *SD* = 3697.63 ms, see ***Figure 3b***).

**Table 2:** Mixed-effects models for pointing error and pointing latency (log-transformed) for participants in the Familiar environment condition.


	POINTING ERROR				POINTING LATENCY			

FOR PARTICIPANTS WHOSE PREFERRED PERSPECTIVE IS THE SAME AS STARTING								

PREDICTOR	R^2^ = .22				R^2^ = .23			
	
	B	SE	t	p	B	SE	t	p

Intercept	9.15	1.74	5.27	<.001	8.57	0.08	113.09	<.001

perspective: Other Orthogonal vs. Preferred/Starting	1.14	1.95	0.59	0.57	**0.19**	**0.08**	**2.38**	**0.04**

**FOR PARTICIPANTS WHOSE PREFERRED PERSPECTIVE IS DIFFERENT THAN STARTING**								

**PREDICTOR**	**R^2^ = .22**				**R^2^ = .31**			
	
	**B**	**SE**	**t**	**p**	**B**	**SE**	**t**	**p**

Intercept	16.60	2.14	7.77	<.001	8.82	0.07	130.23	<.001

perspective: Starting vs. Other Orthogonal	**3.26**	**1.39**	**2.34**	**0.02**	0.08	0.04	1.83	0.08

perspective: Starting vs. Preferred	**5.23**	**1.66**	**3.15**	**<.001**	0.07	0.05	1.48	0.15


*Note*: Each dependent measure is modelled as a function of the centered and contrast coded predictors, using the maximal random effect structure possible. For participants reported preferred orientation was the same as the starting orientation (*N* = 11), perspective type was contrast coded as: preferred/starting = –.5, other orthogonal = .5. For participants reported preferred orientation was different than starting orientation (*N* = 21), perspective type was contrast coded with a simple contrast with “starting” as the reference category. For each fixed effect, we report the unstandardized coefficient and its standard error, along with the associated t-value and p-value, and the overall variance captured by the model (R^2^). Statistically significant fixed effects (at the *alpha* = .05 level) are in bold.

Those participants who reported a preferred perspective that was different from their starting orientation (*N* = 21), in fact performed significantly more accurately from the starting (*M* = 13.85°, *SD* = 17.95°) than the preferred orientation (*M* = 19.56°, *SD* = 22.42°) (see ***Table 2*** and ***Figure 3c***). Performance from the starting orientation was also more accurate than from the other orthogonal orientations (*M* = 16.52°, *SD* = 21.35°). Overall, perspective type influenced pointing error, as inclusion of perspective type significantly improved model fit (χ^2^(2) = 10.38, *p* < .01). Responses were numerically faster from the starting orientation (*M* = 7772.27 ms, *SD* = 5973.05 ms), but did not significantly differ from the preferred (*M* = 8243.45 ms, *SD* = 5397.55 ms) or the other orthogonal orientations (*M* = 8092.59 ms, *SD* = 5432.56 ms). Overall, perspective type did not significantly impact pointing latencies: including perspective type did not significantly improve model fit (χ^2^(4) = 9.39, *p* = .052).

In sum, when reasoning about familiar environments, responses were faster (though not more accurate) from the starting orientation compared to other orthogonal orientations, for participants whose starting orientation coincided with their reported preferred orientation. The starting orientation also exhibited an advantage, in terms of accuracy, for those participants who reported a preferred orientation that was different from their starting orientation.

##### Unfamiliar Environments

**Figure 4:**
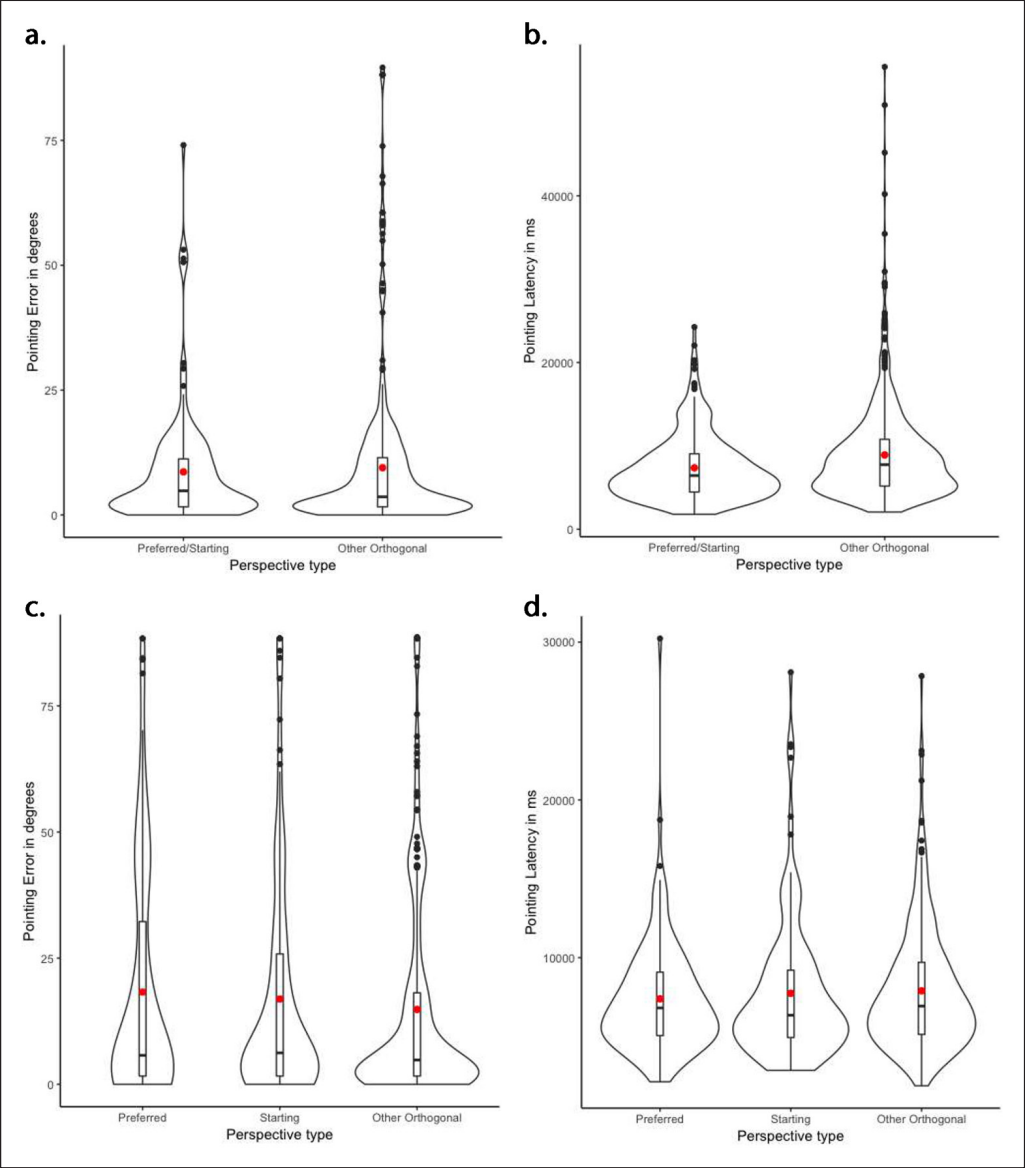
Violin plots representing the distributions of the pointing performance of participants in the Unfamiliar environment condition. **a.** and **b.** illustrate the pointing error and latency of participants whose preferred perspective was the same as the starting perspective (*N* = 21), according to perspective type (preferred/starting vs. other orthogonal perspectives). **c.** and **d.** illustrate the pointing error and latency of participants whose preferred perspective was different from the starting perspective (*N* = 11), according to perspective type (preferred vs. starting vs. other orthogonal perspectives). Boxplots represent the median and quartiles (Q1, Q3), and the red dot represents the mean of each condition. Black dots indicate observations with values greater than Q3 plus 1.5 times the interquartile range.

Following the same approach, we first consider participants in the unfamiliar condition who reported having their starting orientation as their preferred (*N* = 21). Similar to those in familiar environments reporting the same preference, performance in preferred/starting orientation did not differ from other orthogonal orientations in terms of pointing error (preferred/starting: *M* = 13.05°, *SD* = 16.17°; other orthogonal: *M* = 13.70°, *SD* = 18.18°), but it did in terms of pointing latency (preferred starting: *M* = 7365.44 ms, *SD* = 3950.67 ms; other orthogonal: *M* = 8911.71, *SD* = 5723.66; see ***Table 3***). These patterns are illustrated in ***Figure 4a*** and ***4b***.

**Table 3:** Mixed-effects models for pointing error and pointing latency (log-transformed) for participants in the Unfamiliar environment condition.


	POINTING ERROR				POINTING LATENCY			

FOR PARTICIPANTS WHOSE PREFERRED PERSPECTIVE IS THE SAME AS STARTING								

PREDICTOR	R^2^ = .13				R^2^ = 17			
	
	B	SE	t	p	B	SE	t	p

Intercept	13.38	1.57	8.51	<.001	8.86	0.06	44.64	<.001

perspective: Other Orthogonal vs. Preferred/Starting	0.36	1.41	0.25	0.80	**0.16**	**0.04**	**3.91**	**<.001**

**FOR PARTICIPANTS WHOSE PREFERRED PERSPECTIVE IS DIFFERENT THAN STARTING**								

**PREDICTOR**	**R^2^ = .33**				**R^2^ = .10**			
	
	**B**	**SE**	**t**	**p**	**B**	**SE**	**t**	**p**

Intercept	16.59	3.87	4.29	<.001	8.84	0.05	195.61	<.001

perspective: Starting vs. Other Orthogonal	–0.81	2.35	–0.35	0.73	0.04	0.06	0.66	0.52

perspective: Starting vs. Preferred	0.48	2.62	0.18	0.86	–0.03	0.07	–0.41	0.69


*Note*: Each dependent measure is modelled as a function of the centered and contrast coded predictors, using the maximal random effect structure possible. For participants reported preferred orientation was the same as the starting orientation (*N* = 21), perspective type was contrast coded as: preferred/starting = –.5, other orthogonal = .5. For participants reported preferred orientation was different than starting orientation (*N* = 11), perspective type was contrast coded with a simple contrast with “starting” as the reference category. For each fixed effect, we report the unstandardized coefficient and its standard error, along with the associated t-value and p-value, and the overall variance captured by the model (R^2^). Statistically significant fixed effects (at the *alpha* = .05 level) are in bold.

For those participants who reported a preferred perspective that was different from their starting orientation (*N* = 11), performance did not significantly differ across the preferred, starting, and other orthogonal perspectives (see ***Table 3***). Indeed, including perspective type in the models did not significantly improve model fit for neither pointing error (χ^2^(4) = 2.03, *p* = .73) nor pointing latency (χ^2^(4) = 4.26, *p* = .37). The comparable performance across the three perspectives is illustrated in ***Figure 4c*** and ***4d***.

To summarize, for both familiar and unfamiliar environments, participants who reported their preferred orientation to be the same as the starting orientation were faster (but not more accurate) from the starting/preferred orientation than the other orthogonal orientations. Participants who reported a preferred orientation that was different from their starting orientation exhibited an advantage for the starting orientation (in terms of latency) in the familiar condition whereas they exhibited no advantage in the unfamiliar condition.

## DISCUSSION

The present study examined the organizational structure of spatial memories for familiar environments by directly comparing it with the structure of memories for unfamiliar ones. Although participants were, for some perspectives, faster to point to locations in the familiar than the unfamiliar condition, the overall pattern of performance was rather similar across conditions for both pointing accuracy and response time. Specifically, for both familiar and unfamiliar environments, participants pointed faster and more accurately from perspectives aligned with the orthogonal orientations. Their performance was consistent with the sawtooth pattern reported in past studies with small-scale unfamiliar environments (Mou & McNamara, 2002; Shelton & McNamara, 2001; Street & Wang, 2014) and large-scale familiar environments (Marchette et al., 2011). Thus, extending the results of past studies, our findings indicate that memories for small-scale familiar environments, which are typically encoded solely via direct experience as opposed to map learning or a combination of inputs, are also orientation dependent.

Although a sawtooth pattern was observed in both environment conditions here, there was a notable difference in performance, as indicated by the numerical differences in pointing latency and accuracy across the four orthogonal perspectives. In the unfamiliar condition, there was a clearer advantage for the 0°–180° axis compared to the familiar condition. In the familiar condition, performance was about equal across all four orientations that were aligned with the geometry of room. The advantage for a single reference axis in the unfamiliar condition, along with the presence of a sawtooth pattern of performance, replicates the results of past studies on spatial memory that require participants to commit a layout to memory just prior to testing (e.g., Mou & McNamara, 2002; Kelly et al., 2007). In these studies, performance is typically best for one orientation and the one opposite from it, compared to remaining orientations. In the familiar condition, the presence of a sawtooth pattern replicates the findings of Marchette et al. (2011). However, in contrast with Marchette et al. (2011) who found an advantage for a single reference direction, here performance was about equal for all 4 orientations aligned with the geometric axes of the room. One possible explanation for this outcome is that our participants had more experience with the environment and from multiple perspectives than participants did in Marchette et al. (2011), which allowed them to easily adopt and locate objects from those perspectives. Perhaps participants’ extensive experience of their room facilitated the retrieval of information about their imagined orientation in space from an egocentric representation, as well as from an allocentric representation. Thus, performance in the familiar condition may have benefited from the flexible switching between egocentric and allocentric representations, as suggested by Bird and Burgess (2008).

Alternatively, it could be that the advantage of a reference direction in the familiar condition was masked by the influence on performance of the starting orientation. It should be noted that in several studies on spatial memory for unfamiliar environments, the viewpoint from which a layout is studied is held constant (with participants not allowed to move away from it) and manipulated to match or mismatch other cues such as the environment geometry or social factors (e.g., Galati & Avraamides, 2015; Kelly et al., 2018). When no strong cues prime another perspective, the study viewpoint typically determines the reference direction from which the layout is represented in memory (see Galati & Avraamides, 2013, for evidence and a discussion on the influence of multiple cues on spatial memory). Even in studies in which participants are allowed to move in order to study the array from any viewpoint, the starting orientation (i.e., the first viewpoint from which the layout is experienced) is still found to influence performance. That is, when no salient environmental cues are present and no instructions are given to memorize the layout from a different perspective, the first viewpoint is associated with the best performance, leading researchers to conclude that it was used as the reference orientation for representing information in memory (e.g., Avraam, Hatzipanayioti, & Avraamides, 2020). In the present study, we manipulated the starting orientation by having participants in the unfamiliar condition study the room in VR starting from one of the four orientations that were aligned with the geometric structure. Similarly, in the familiar condition we showed participants the objects from their room starting from one of the four orthogonal perspectives. This manipulation may have masked – partially or fully – the advantage of a reference direction. Indeed, as our results showed, this starting perspective has influenced participants’ behavior in a number of ways.

First, when asked after the conclusion of the pointing trials to imagine themselves back in the environment and report the perspective they had spontaneously adopted, many participants reported being oriented along the starting perspective. This, however, was overwhelmingly the case in the unfamiliar condition where 2/3 of participants reported having assumed the starting orientation, compared to 1/3 of participants in the familiar condition. This pattern was expected, as participants in the familiar condition lived in the room for at least a year and thus had a more robust memory representation, making them more likely to have a “default” perspective already and be less influenced to report a different perspective–cued during the experiment–as their preferred.

Second, the starting orientation seemed to exert an influence on pointing performance as well. This influence was observed to a greater extent in the familiar than in the unfamiliar condition. In the familiar condition–but also in the unfamiliar condition–participants pointed faster from the starting than other orientations when the starting was the reported preferred. More importantly, in the familiar condition, even when participants reported a different preferred orientation than the starting orientation, they still pointed more accurately from the starting than other orthogonal orientations, including the preferred. Thus, overall, the starting orientation influenced performance in the familiar condition regardless of whether participants reported it as their preferred orientation when asked to imagine themselves back in the room after testing.

This finding–that participants’ self-reported preferred orientation was not associated with better performance than the remaining orthogonal orientations–is in line with past results from Yerramsetti et al. (2013). In one condition of that study, participants performed undirected JRDs: they were asked to point from one campus landmark to another without given a facing orientation. Participants were consistent – both across to one another and within themselves across trials involving the same building – in selecting the same assumed orientation; however, this spontaneous orientation did not match with the perspective yielding best performance when participants were tested with traditional JRDs, in which an imagined perspective was stated in each trial. Similarly, in our study, the self-reported preferred orientation did not unequivocally lead to the best performance. In fact, in our familiar condition participants pointed more accurately from the starting orientation than other orientations including the preferred orientation.

As Yerramsetti and colleagues (2013) suggested, participants’ spontaneous preferred orientation was most likely determined by dominant visual cues and was the perspective permitting the broadest field of view from the building. In our study, participants were also influenced by dominant visual cues of the environment. Most participants in the familiar condition reported facing the window as their preferred perspective: this perspective allowed for the broadest field of view within the room as it was aligned with the elongated axis of the rectangular room. Notably, the opposite perspective with the window at one’s back also allowed for an equally broad view of the room. The preference for the orientation towards than away from the window in the familiar condition could have been due to the window being larger and more salient than the other objects or that the view coincided with the first view participants got each time they entered the room.

That the starting orientation had an influence on performance is a notable finding on various grounds. First, in the absence of prior knowledge about the environment, the starting orientation became the preferred orientation for most participants in the unfamiliar condition. Second, participants in the familiar condition who reported a preferred orientation different from the starting were actually more accurate to point to objects from the starting than the reported preferred. This finding is in line with Yerramsetti et al. (2013), underscoring that perceptual and experiential properties of a space may define a preferred orientation that is not necessarily the most accessible in a spatial perspective-taking task. Still, a question persists: why would the starting perspective yield an advantage for performance if it’s not adopted as the preferred reference direction for representation?

A plausible explanation is that the advantage of the starting orientation reflects a priming effect during retrieval. That is, the first view participants got of the environment (either in VR or on the desktop screen) served to activate a spatial representation from that particular viewpoint. This explanation is in line with past findings showing that prior activation of a perspective through mental travel in a familiar environment influences the orientation of sketches produced about the environment (Basten, Meilinger, & Mallot, 2012). In that study, passersby at a cafeteria were asked to draw a sketch of a well-known square in the city of Tübingen in Germany after imagining walking along a route crossing the square. Results showed that most participants drew the square east-up or west-up depending on the orientation of their imaginal walk. Notably, in a control condition without mental travel most participants drew the square with a south-up orientation. These findings suggest that situational context that gets activated, even by imagination, can influence the orientation from which the spatial information is externalized. Similarly, in our study, the visual context provided by the starting orientation could have primed that orientation, rendering it more accessible in the JRD task.

Given that in our experiment the starting orientation influenced performance more in the familiar than the unfamiliar condition (when distinct from the preferred perspective) may suggest that the presence of a consolidated spatial representation is needed for priming to occur. For unfamiliar environments, the starting orientation showed facilitation (in terms of pointing latency) only when it was adopted as the preferred; when it was not, it did not show an advantage. Future work can examine whether our proposal holds–that perspectives can be robustly primed only in consolidated representations: this can be examined by investigating the time course with which an advantage of the starting orientation emerges as people become increasingly familiar with an environment.

Another question that arises is whether the starting orientation would still influence performance if it were not aligned with the geometric structure of the room. Past research indicates that when multiple conflicting cues are available during encoding, participants weigh these cues to select the reference orientation or axes from which to represent spatial information (see Galati & Avraamides, 2013 for a discussion). As a result, it is possible that if egocentric experience was misaligned to the room geometry during encoding, at least some participants in the unfamiliar condition would represent the room environment based on their egocentric experience than the room geometry. That said, we consider this rather unlikely given results from prior studies showing that salient environmental cues generally dominate egocentric experience (e.g., Mou, Liu, & McNamara, 2009; Mou & McNamara, 2002) in establishing the organization of spatial memory. However, it is still possible, that in both unfamiliar and familiar conditions, an advantage for a misaligned starting orientation would result from post-encoding priming processes. This is an interesting empirical question that could also be explored in future studies.

In sum, the results from our study show that memories for familiar environments are somewhat different than those for unfamiliar environments. Although they exhibit the same organizational principles that yield a sawtooth pattern of performance when reasoning from imagined perspectives, they also seem to rely less on a single reference orientation. We found that memories for familiar environments are more flexibly accessible from other orthogonal perspectives, perhaps due to easier mental transformations afforded by the well-learned environment and/or the involvement of egocentric representations. Of course, our results characterize small-scale environments which are encoded on the basis of a single reference frame. As already noted, large-scale navigable environments may be represented hierarchically with parts of the environment organized around different local reference frames and the spatial relations among the parts represented in a global reference frame. As such representations rely on knowledge that is acquired over time, differences in spatial reasoning about large-scale familiar and unfamiliar environment may depend on the amount of familiarity at the time of test (e.g., whether a complete hierarchical representation is in place or not). Finally, our results underscore that the accessibility of spatial information from well-established long-term memories is influenced by post-encoding situational factors, such as which view is presented first. Probing someone to begin viewing a familiar environment from a particular perspective can prime the accessibility of spatial information from that perspective, and even temper the effect of their reported perspective preference. This result cautions researchers to be careful when making inferences about representation from the pattern of accuracy and reaction time in spatial perspective-taking.

## Footnotes

At the time the picture was taken participants had already signed up to participate in the study and they were told that the picture would be used for the testing. However, they were not told that testing would entail recalling the locations of objects in their rooms.
